# Cost of illness of the Cervical Cancer of the uterus in Japan - a time trend and future projections

**DOI:** 10.1186/s12913-015-0776-5

**Published:** 2015-03-15

**Authors:** Eijiro Hayata, Kanako Seto, Kayoko Haga, Takefumi Kitazawa, Kunichika Matsumoto, Mineto Morita, Tomonori Hasegawa

**Affiliations:** Department of Obstetrics and Gynecology, Toho University Omori Medical Center, 6-11–1 Omori-nishi, Ota-ku, Tokyo 143-8541 Japan; Department of Social Medicine, Toho University School of Medicine, 5-21–16 Omori-nishi, Ota-ku, Tokyo 143-8540 Japan

**Keywords:** Cost of illness (COI), Economic burden of disease, Cervical cancer, Health economics, Health policy

## Abstract

**Background:**

Cervical cancer is associated with high morbidity and mortality rates among young women in Japan. The objective of this study was to assess and project the economic burden associated with cervical cancer in Japan and identify factors affecting future changes in this burden on society.

**Methods:**

Utilizing government-based statistical nationwide data, we used the cost of illness (COI) method to estimate the COIs for 1996, 1999, 2002, 2005, 2008, and 2011 to make predictions for 2014, 2017, and 2020. The COI comprised direct and indirect costs (morbidity and mortality costs).

**Results:**

The COI was estimated to have increased by 66% from 96.1 billion yen in 1996 to 159.9 billion yen in 2011. The number of deaths increased, but the proportion of those aged ≥65 years as a percentage of all deaths remained mostly unchanged, with no increase in the average age at death. The mortality cost per person was estimated to have increased (31.5 million yen in 1996 vs. 43.5 million yen in 2011). Assuming that the current trend in health-related indicators continues, the COI is predicted to temporarily decrease in 2014, followed by almost no change in 2020 (the estimated COI is 145.3–164.6 billion yen). The mortality cost per person is predicted to remain almost unchanged (39.4–46.3 million yen in 2020).

**Conclusions:**

The fact that the life expectancy of affected individuals is not being prolonged and that the mortality in young individuals with a high human capital value is not decreasing may contribute to future sustainment of the COI. We believe that the results of the present study are applicable to discussions of disease control priorities.

**Electronic supplementary material:**

The online version of this article (doi:10.1186/s12913-015-0776-5) contains supplementary material, which is available to authorized users.

## Background

Cervical cancer (ICD-10 code: C53) affected 9,794 Japanese women in 2008 and resulted in 2,737 deaths in 2011, making it the 9th most common type of cancer in terms of morbidity and the 13th most common cancer in terms of mortality in women in these years [1]. However, when limited to women aged <40 years, it is the second most common cancer following breast cancer in terms of morbidity and the third most common cancer following breast and stomach cancer in terms of mortality [1].

Because cervical cancer is associated with high morbidity and mortality rates among relatively young women, its economic burden is predicted to change as women’s participation in society increases. However, few studies have estimated the economic burden of cervical cancer; most reports have estimated only direct treatment costs and performed cost-effectiveness analyses of specified technologies [2-4]. No such long-term studies have been conducted, and none have made future predictions. The objectives of the present study were to assess the economic burden of cervical cancer over time, make future predictions based on these observations, and identify the factors that drive the social burden of cervical cancer.

## Methods

### Analysis method

We used the cost of illness (COI) method to examine the economic burden of cervical cancer. Government-based statistical data and the COI method proposed by Rice *et al.* were used to make COI estimates [5-12]. The COI includes both direct cost (DC) and indirect cost (IC); IC includes morbidity cost (MbC) and mortality cost (MtC). The COI is calculated using following equation:

$$ \mathrm{C}\mathrm{O}\mathrm{I}=\mathrm{D}\mathrm{C}+\mathrm{M}\mathrm{b}\mathrm{C}+\mathrm{M}\mathrm{t}\mathrm{C} $$

DCs are medical costs that arise directly as a result of disease, such as costs of treatment, hospitalization, testing, and drugs. We calculated the annual medical costs in the present study from the total medical expenses using the Survey of National Medical Care Insurance Services [13]. This survey only contains data for calculating DCs up to mid-level injuries and disease classifications such as uterine cancer (C53–55); it does not contain lower-level injury and disease classifications, such as cervical cancer of the uterus (C53). Therefore, we calculated DCs by proportionally distributing medical expenses based on the estimated number of patients (ENP) for cervical cancer (C53), endometrial cancer (C54), and other uterine cancers (C55) from the Patient Survey of the Ministry of Health, Labour and Welfare [14]. Thus, DC was calculated using the following equation:

$$ \mathrm{D}\mathrm{C}=\mathrm{D}\mathrm{C}\left(\mathrm{C}53-55\right)\times \mathrm{C}\left(\mathrm{C}5\mathrm{C}53\right)/\mathrm{E}\mathrm{N}\mathrm{P}\left(\mathrm{C}53-55\right) $$

ICs are opportunity costs lost as a result of disease or death. MbC and MtC were calculated using the following equations:

$$ \mathrm{M}\mathrm{b}\mathrm{C}=\mathrm{TOVy}\times \mathrm{OLVd}/2+\mathrm{T}\mathrm{H}\mathrm{D}\times /\mathrm{L}\mathrm{V}\mathrm{d}\ \mathrm{and} $$

$$ \mathrm{M}\mathrm{t}\mathrm{C}=\mathrm{N}\mathrm{D}\mathrm{y}\times \mathrm{L}\mathrm{V}\mathrm{l}, $$

where TOVy is the total person-days of outpatient visits, LVd is the 1-day labor value per person, THD is the total person-days of hospitalization, NDy is the number of deaths, and LVl is the lifetime labor value per person.

The TOVy and THD according to 5-year age groups were calculated based on the above-mentioned Patient Survey [14]. The labor values were calculated according to 5-year age groups using the Basic Survey on Wage Structure [15], Labor Force Survey [16], Estimates of Monetary Valuation of Unpaid Work [17], and Evaluations of Domestic Labor [18], each of which is associated with a specific government office. The LVl was calculated by summing the income that could have been earned in the future if death had not occurred. We calculated the MbC by assuming a 1-day labor value loss for 1 hospitalized day and a half-day labor value loss for one outpatient visit. We used the number of deaths by cervical cancer according to 5-year age groups from the Vital Statistics of the Ministry of Health, Labour and Welfare [19].

LVd and THD were calculated as follows:

$$ \mathrm{L}\mathrm{V}\mathrm{d}=\left(\mathrm{I}\mathrm{y}+\mathrm{ULVy}\right)/365\ \mathrm{and} $$

$$ \mathrm{T}\mathrm{H}\mathrm{D}=\mathrm{H}\mathrm{P}\mathrm{y}\times \mathrm{ALOS}, $$

where Iy is the annual income per person [15], ULVy is the annual monetary valuation of unpaid work per person [17,18], HPy is the annual number of hospitalized patients [14], and ALOS is the average length of stay [14]. The future labor value was adjusted to a present value using a 3% discount rate because 3% is widely used as discount rate in developed countries such as Japan and the United States, where application of the COI method is popular.

### COI estimates over time

To examine changes over time, we first estimated the COIs for 1996, 1999, 2002, 2005, 2008, and 2011 using available data. The effect of introducing specific therapeutic techniques was not examined in this study.

Next, to make future predictions, we estimated the COIs for 2014, 2017, and 2020 using the two methods described below.

The first method involved fixing health-related indicators (mortality rate, average number of outpatient visits, average number of hospitalizations, average length of stay, and medical fees per examination) at the 2011 values and making estimates assuming changes in Japan’s population and age composition (hereafter referred to as fixed-model estimation). The data regarding medical fees per examination were obtained from the Patient Survey [14]. Using 2011 as the standard year, we first calculated the mortality rate, average number of outpatient visits, average number of hospitalizations, and average length of stay according to 5-year age groups in 2011. These data were multiplied by the estimated populations for 2014, 2017, and 2020 according to 5-year age groups to obtain the predicted mortality rate, average number of outpatient visits, average number of hospitalizations, and average length of stay for each year. Using data from 2011, the estimated average length of stay, mean life expectancy, and labor value were used to estimate MbC and MtC. DCs were calculated by multiplying the rate of increase in outpatients and inpatients from 2011 to 2014, 2017, and 2020 by the outpatient and inpatient costs for 2011.

The second method involved making estimates assuming continued trends in health-related indicators as well as changes in population and age compositions. After calculating approximation curves (logarithmic and linear approximations) for each item using data after 1996, we obtained figures for 2014, 2017, and 2020. We then calculated the annual rate of increase in medical fees per examination (outpatient and inpatient) [14] from 1996 to 2011 and corrected this increase by multiplying it by outpatient and inpatient costs for 2014, 2017, and 2020. When making future predictions using this method, estimates varied depending on which approximation with which they were calculated. Three patterns of estimates were made: estimates made from logarithmic approximations of all items (logarithm-model estimation), estimates made from linear approximations of all items (linear-model estimation), and estimates made from approximations of items with higher determination coefficients (mixed-model estimation). Logarithmic and linear estimates would produce different annual trends depending on the item; thus, single estimates are likely to produce overestimated or underestimated values. We therefore compared determination coefficients within the same age group in both the logarithmic and linear approximation curves and performed a mixed estimate using the curve with the higher coefficient in each age group. Mixed-model estimation was considered the most likely estimates in this study. The elements used for calculation of the predicted future COI are shown in Table 1.

**Table 1:** **Elements used for calculation of predicted future cost of illness**

**Model**	**Item**	**Elements used for calculation**	**Fixed or Varied**
Fixed model	Number of deaths	Mortality rate	Fixed
		The population estimates	Varied
	Direct cost	The expenses of outpatient visit and hospitalization	Fixed (Calculated using the unit cost in 2011)
		Medical fees per examination	Fixed
		Total person-days of outpatient visit	Varied
		Total person-days of hospitalizations	Varied
	Morbidity cost	Average number of outpatients	Fixed
		Average number of hospitalizations	Fixed
		Average length of stay	Fixed
		The population estimates	varied
		Labor-value	Fixed
	Mortality cost	Number of deaths	Varied
		Life expectancy	Fixed
		Labor-value	Fixed
		Discount rate: 3%	Fixed
・Logarithm model	Number of deaths	Mortality rate	Varied (Calculated using the trend line formula)
・Linear model		The population estimates	Varied
・Mixed model	Direct cost	The expenses of outpatient visit and hospitalization	Fixed (Calculated using the unit cost in 2011)
		Medical fees per examination	Varied
		Total person-days of outpatient visit	Varied
		Total person-days of hospitalization	Varied
	Morbidity cost	Average number of outpatient visits	Varied (Calculated using the trend line formula)
			(minimum value: the previous value before 0)
		Average number of hospitalizations	Varied (Calculated using the trend line formula)
			(minimum value: the previous value before 0)
		Average length of stay	Varied (Calculated using the trend line formula)
			(minimum value: 7.8 days)
		The population estimates	Varied
		Labor-value	Fixed
	Mortality cost	Number of deaths	Varied
		Life expectancy	Fixed
		Labor-value	Fixed
		Discount rate: 3%	Fixed

Fixed: The value for 2011 was used. Varied: The values for 2014, 2017, and 2020 were calculated based on the trend line.

Source of medical fees per examination: Patient Survey [14].

Source of average length of stay: Patient Survey [14].

Data from 2011 were used to determine the average life expectancy and the labor value. Additionally, the Population Estimates [20] by the Ministry of Internal Affairs and Communications were used for 1996, 2002, 2005, 2008, and 2011 population statistics, while the Population Projections for Japan [21] by the National Institute of Population and Social Security Research were used to make estimates for 2014, 2017, and 2020.

For estimates using approximation curves, some future predicted values may be <0; however, these are unlikely to reflect actual clinical conditions. Therefore, we defined “minimum values” in the present study as described below. For mortality and the numbers of outpatients and inpatients per person, the minimum value was set as the value from the year prior to that in which the estimate was <0. When examined according to age groups, we assumed that the value from the year prior to that when the estimate was <0 would be maintained thereafter and thus applied this value when estimates for 2014, 2017, and 2020 fell to <0. The minimum value for the average length of stay was set at 7.8 days, which is the average in 28 countries (2010) reporting data on neoplasms; this was based on the average length of stay by diagnostic categories in the OECD Health Data 2013 (statistics and indicators). According to the 2011 Patient Survey [14], the average length of stay for cervical cancer (17.8 days) did not differ greatly from the overall mean hospitalization period for malignant neoplasms (20.6 days); thus, we set the mean value for neoplasms in this study as the minimum length of stay for cervical cancer and applied this to age groups with hospitalization periods of <7.8 days in the estimates for 2014, 2017, and 2020. This study used only aggregated and published nationwide data that are freely available online; no humans or animals were used. In Japan, no institutional review board approval is required for this type of study [22].

## Results

### COI estimates for 1996, 1999, 2002, 2005, 2008, and 2011

The COI was estimated at 96.1 billion yen in 1996, 110.6 billion yen in 1999, 131.7 billion yen in 2002, 133.4 billion yen in 2005, 145.5 billion yen in 2008, and 159.9 billion yen in 2011, indicating an increasing trend. Comparison of estimates from 1996 and 2011 revealed that the COI had increased by 66%. The number of deaths increased, but the proportion of those aged ≥65 years as a percentage of all deaths remained unchanged, with no increase in the average age at death. No change was observed in the fatality rate. The MtC per person calculated by dividing the MtC by the number of deaths followed an increasing trend (Table 2). Additional details of results (TOVy, THD, and LVl) are provided in Additional file 1.

**Table 2:** **Time trend of cost of illness of cervical cancer**

	**1996**	**1999**	**2002**	**2005**	**2008**	**2011**
Population (thousand person)	125,864	126,686	127,435	127,768	127,692	127,799
[% of 65 years or older]	15.1%	16.7%	18.5%	20.2%	22.1%	23.1%
Number of cervical cancer deaths (person)	2,219	2,260	2,443	2,465	2,486	2,737
[% of 65 years or older]	51.7%	51.1%	47.7%	48.2%	47.7%	49.6%
Average age of death (year)	64.1	63.6	62.9	63.6	63.4	64.0
Crude incidence rate (per 100 thousand, female)	12.1	10.7	13.5	13.0	15.0	NA
Crude mortality rate (per 100 thousand, female)	3.49	3.53	3.79	3.82	3.85	4.23
Fatality rate (female)	0.29	0.33	0.28	0.29	0.26	NA
Direct cost (billion yen)	15.8	26.0	20.1	25.3	33.6	31.0
Morbidity cost (billion yen)	10.4	10.1	12.8	10.3	10.6	9.9
Mortality cost (billion yen)	69.9	74.5	98.8	97.8	101.3	119.0
[% of 65 years or older]	16.6%	15.4%	15.8%	16.9%	15.2%	17.5%
Mortality cost per person (million yen)	31.5	33.0	40.4	39.7	40.7	43.5
COI (billion yen)	96.1	110.6	131.7	133.4	145.5	159.9

Source of population: Ministry of Internal Affairs and Communications, Population Estimates.

Source of number of cervical cancer deaths: Vital Statistics.

Average age of death: Calculated according to the number of deaths, age (5-year age grades), and cause of death in Vital Statistics.

Source of crude morbidity rate and crude mortality rate: Center for Cancer Control and Information Services, National Cancer Center, Japan.

Fatality rate: Calculated by dividing the crude mortality rate by the crude morbidity rate.

NA: not available.

### COI estimates for 2014, 2017, and 2020 (fixed-model estimation)

The COI was estimated at 159.5 billion yen for 2014, 158.9 billion yen for 2017, and 156.8 billion yen for 2020, with almost no change. DC, MbC, and MtC were all predicted to remain the same (Table 3). The predicted number of deaths showed an increasing trend and is expected to increase by 7.9% from 2011 to 2020. The proportion of deaths in individuals aged ≥65 years is expected to increase (49.6% in 2011, 56.8% in 2020), as is the average age at death (64.0 years in 2011, 66.3 years in 2020). The MtC of those aged ≥65 years as a percentage of the total is expected to be 20.8% in 2020, showing a decreasing trend in the MtC per person.

**Table 3:** **Future prediction of cost of illness**

**Model**	**Item**	**2011**	**2014**	**2017**	**2020**
	Estimated population (thousand person)	127,799	126,949	125,739	124,223
	[% of 65 years or older]	23.1%	26.1%	28.0%	29.1%
Fixed model	Number of cervical cancer deaths (person)	2,737	2,816	2,900	2,953
	[% of 65 years or older]	49.6%	53.0%	55.5%	56.8%
	Average age of death (year)	64.0	64.8	65.6	66.3
	Direct cost (billion yen)	31.0	31.1	31.0	30.8
	Morbidity cost (billion yen)	9.9	9.9	9.8	9.7
	Mortality cost (billion yen)	119.0	118.5	118.1	116.3
	[% of 65 years or older]	17.5%	19.3%	20.5%	20.8%
	Mortality cost per person (million yen)	43.5	42.1	40.7	39.4
	COI (billion yen)	159.9	159.5	158.9	156.8
Linear model	Number of cervical cancer deaths (person)	2,737	2,611	2,598	2,553
	[% of 65 years or older]	49.6%	48.3%	47.4%	45.0%
	Average age of death (year)	64.0	63.1	62.8	62.3
	Direct cost (billion yen)	31.0	23.1	20.8	22.2
	Morbidity cost (billion yen)	9.9	6.7	5.5	5.0
	Mortality cost (billion yen)	119.0	116.1	117.7	118.1
	[% of 65 years or older]	17.5%	16.4%	15.5%	13.9%
	Mortality cost per person (million yen)	43.5	44.5	45.3	46.3
	COI (billion yen)	159.9	145.9	144.0	145.3
Logarithm model	Number of cervical cancer deaths (person)	2,737	2,714	2,772	2,812
	[% of 65 years or older]	49.6%	52.6%	54.0%	54.6%
	Average age of death (year)	64.0	64.4	64.8	65.3
	Direct cost (billion yen)	31.0	36.9	40.5	43.1
	Morbidity cost (billion yen)	9.9	9.6	8.9	8.1
	Mortality cost (billion yen)	119.0	113.3	114.0	113.4
	[% of 65 years or older]	17.5%	19.0%	19.4%	19.2%
	Mortality cost per person (million yen)	43.5	41.7	41.1	40.3
	COI (billion yen)	159.9	159.8	163.4	164.6
Mixed model	Number of cervical cancer deaths (person)	2,737	2,604	2,584	2,549
	[% of 65 years or older]	49.6%	49.7%	49.3%	48.0%
	Average age of death (year)	64.0	63.5	63.3	63.2
	Direct cost (billion yen)	31.0	27.7	26.6	28.4
	Morbidity cost (billion yen)	9.9	7.8	6.8	6.4
	Mortality cost (billion yen)	119.0	113.3	113.5	113.0
	[% of 65 years or older]	17.5%	17.5%	17.0%	16.0%
	Mortality cost per person (million yen)	43.5	43.5	43.9	44.3
	COI (billion yen)	159.9	148.8	146.9	147.8

Source of estimated population, 2011: Ministry of Internal Affairs and Communications, Population Estimates 2014, 2017, and 2020: National Institute of Population and Social Security Research, Population Statistics of Japan.

### COI estimates for 2014, 2017, and 2020 (linear-, logarithm-, and mixed-model estimations)

According to the linear-model estimation, the COI was predicted to temporarily decrease to 145.9 billion yen in 2014, after which it is predicted to remain mostly unchanged at 144.0 billion yen in 2017 and 145.3 billion yen in 2020. This implies a 9.1% decrease from 2011 to 2020. According to the logarithm-model estimation, the COI is estimated to remain mostly unchanged at 159.8 billion yen in 2014, 163.4 billion yen in 2017, and 164.6 billion yen in 2020. The predicted number of deaths is predicted to decrease in the linear-model estimation and increase in the logarithm-model estimation (Table 3).

The linear-model estimation showed that DC, MbC, and MtC will decrease temporarily in 2014, after which they will remain mostly unchanged until 2020. The logarithm-model estimation predicts that DC will increase, whereas MbC and MtC will decrease.

According to the mixed-model estimation, the COI was estimated to temporarily decrease to 148.8 billion yen in 2014, after which it will remain mostly unchanged at 146.9 billion yen in 2017 and 147.8 billion yen in 2020. The rate of decrease from 2011 to 2020 is estimated at 7.6%. The number of deaths and the MbC are estimated to decrease over time from 2011, and DC, MtC, and the COI will temporarily decrease in 2014, after which they are estimated to remain mostly unchanged until 2020. Although mixed-model estimation is predicted to be below fixed-model estimation for all items, they are predicted to exceed linear estimates. The proportion of deaths in those aged ≥65 years is expected to decrease to 48.0% in 2020, and the average age at death will decrease to 63.2 years. Furthermore, the MtC per person is predicted to increase to 44.3 million yen, and the proportion of those aged ≥65 years as a percentage of MtC is estimated to decrease to 16.0%.

**Figure 1:**
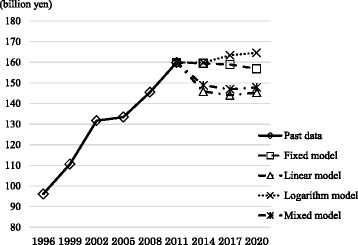
**Time trends of cost of illness by prediction models.**

**Figure 2:**
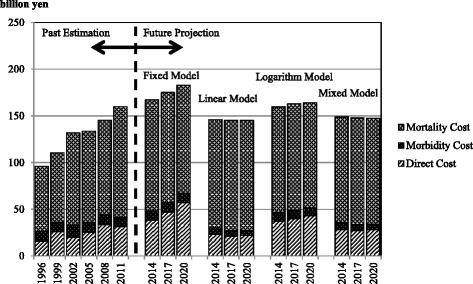
**Cost of illness projection with cost elements.**

The COI over time from 1996 is shown in Figures 1 and 2. The results suggest that the COI of cervical cancer will temporarily decrease and then remain mostly unchanged.

## Discussion

The COI increased from 1996 to 2011. Based on our future predictions, the COI is estimated to remain unchanged according to fixed-model estimates. However, assuming that current trends in health-related indicators continue, linear-model estimates show that the COI will temporarily decrease in 2014, followed by almost no change. On the other hand, logarithmic-model estimates suggest that the trends will remain mostly unchanged from 2011. Mixed-model estimates, which are considered the most reliable, show that despite the decreased number of deaths, the COI will remain mostly unchanged from 2014, with DC and MtC following the same trend. The main model used in our analysis was the mixed model. The fixed model was a reference. The linear and logarithm models provide low-end and high-end estimation, respectively, and they can be regarded as sensitivity analyses that show the robustness of the mixed model.

Factors that influenced the increase in the COI from 1996 to 2011 were greater DC and MtC. In contrast, MbC remained mostly unchanged. The medical fees per examination of patients with uterine cancer increased by 5.3% annually, which is believed to be linked to greater DC due to the increasing trend in the estimated number of patients. The increase in medical fees per examination is likely influenced by recent advancements in medical care. On the other hand, the mean length of hospitalization has shown a consistent downward trend, which may explain the lack of the increase in MbC, canceling out the increase in medical fees and opportunity costs lost as a result of hospitalization. The increase in MtC is important considering the age at death of patients with cervical cancer and the impact of aging in Japan. The average age at death has remained mostly unchanged due to a twin-peak pattern of increasing numbers of deaths at younger ages (30–64 years) and older ages (≥75 years) [19]. In general, because the fatality rate in the older population is high, aging is a factor that increases the number of deaths and MtC. However, aging also decreases MtC because older individuals have relatively lower human capital value. Thus, the impact of aging on MtC is relatively small. However, increased numbers of deaths of young people contributes significantly to the increase in MtC. The annual labor value per person is increasing due to the increase in the labor force participation rate among young women and increasing annual incomes, which reflect the social advancement of women. The increase in the number of deaths and labor value per person among young women is believed to be a significant factor affecting the increase in MtC (Table 4). In a previous report [23] in which the COI of stomach cancer was estimated using the same method described herein, the COI of stomach cancer decreased by 13.9% from 1996 to 2008, which can be attributed to decreased MbC and MtC. Although the number of stomach cancer-related deaths remained unchanged, the average age at death increased, which may have led to a decrease in the MtC per person and an overall decrease in MtC. Additionally, the decreased number of outpatients and inpatients and shortened hospitalization may have reduced MbC. The present study shows a COI trend opposite to that in the above-mentioned study. COI trends differ according to patient attributes and disease features, even for the same solid cancer. Comparison of each factor influencing the COI would allow for evaluation of the characteristics of the economic burden of each disease, which would contribute to the design of future health policies.

**Table 4:** **Time trend of number of cervical cancer deaths, labor force participation rate, annual income, and annual labor value**

		**25–29**	**30–34**	**35–39**	**40–44**	**45–49**	**50–54**	**55–59**	**60–64**	**65–69**	**70–74**	**75–79**	**80–84**	**85-**
Number of cervical cancer deaths (person) [19]	1996	16	44	68	105	209	195	210	224	256	233	251	208	200
	2011	19	68	118	202	219	221	228	304	231	220	281	270	356
	changing rate	18.8%	54.5%	73.5%	92.4%	4.8%	13.3%	8.6%	35.7%	-9.8%	-5.6%	12.0%	29.8%	78.0%
Labor force participation rate (%) [16]	1996	67.9	54.8	60.8	69.5	71.6	66.9	58.1	39.0	27.0	10.1	0	0	0
	2011	77.0	67.5	67.0	71.2	75.7	72.6	64.0	45.7	27.6	8.6	0	0	0
	changing rate	13.4%	23.2%	10.2%	2.4%	5.7%	8.5%	10.2%	17.2%	2.2%	-14.9%	0.0%	0.0%	0.0%
Annual income per person (thousand yen) [15]	1996	1817	1578	1802	2010	2036	1887	1555	969	664	248	0	0	0
	2011	2176	2050	2132	2321	2457	2323	1942	1173	678	230	0	0	0
	changing rate	19.8%	29.9%	18.3%	15.5%	20.7%	23.1%	24.9%	21.1%	2.1%	-7.3%	0.0%	0.0%	0.0%
Annual labor value per person (thousand yen)	1996	3584	4064	4288	4051	4078	3745	3414	2837	2297	1684	1181	742	304
	2011	3493	4339	4900	5087	5004	4681	4305	3223	2608	2301	1837	1513	800
	changing rate	-2.5%	6.8%	14.3%	25.6%	22.7%	25.0%	26.1%	13.6%	13.5%	36.6%	55.5%	103.9%	163.2%

Source of number of cervical cancer deaths: Vital Statistics [19].

Source of labor force participation rate: Labor Force Survey, Ministry of Internal Affairs and Communications [16].

Source of annual income per person: Basic Survey on Wage Structure, Ministry of Health, Labour and Welfare [15].

Annual labor value per person: Calculated according to annual income per person, labor force participation rate, and estimates of monetary valuation of unpaid work.

According to our future predictions (fixed-model estimation), the number of deaths will increase, but the COI, DC, MbC, and MtC are estimated to remain unchanged. In particular, MtC is estimated to decrease to no more than 2.3% irrespective of the fact that the number of deaths will increase by 7.8% from 2011 to 2020. The increase in the proportion of deaths in those aged ≥65 years (49.6% in 2011, 56.8% in 2020), the increase in the average age at death (64.0 in 2011, 66.3 in 2020), and the decrease in the MtC per person as a result of the reduced human capital value (43.5 million yen in 2011, 39.4 million yen in 2020) are estimated to be offset by the pressure of increased MtC from the increased number of deaths.

According to the mixed-model estimation, the COI is estimated to temporarily decrease in 2014, followed by almost no change thereafter. The same trend is expected for the number of deaths, DC, MbC, and MtC. The proportion of deaths in those aged ≥65 years will decrease (49.6% in 2011, 48.0% in 2020), as will the average age at death (64.0 in 2011, 63.2 in 2020), and the MtC per person will increase (43.5 million yen in 2011, 44.3 million yen in 2020). The previously mentioned COI estimates of stomach cancer [22] predict decreases in the number of deaths, DC, MbC, MtC, and overall COI. In particular, a 25.1% decrease in the number of deaths and a 55.6% decrease in MtC from 2008 to 2020 are likely factors influencing these estimates. The increase in the average age at death and the sharp decline in the number of deaths among young people with a high human capital value are predicted to be linked to decreased MtC. Compared with stomach cancer, no increase in the average age at death can be observed for cervical cancer, and the lack of the reduction in the number of deaths among young women with high human capital value is a likely factor preventing future reductions in the COI. COI may rather increase in the future because of the promotion of women’s participation in society and the increase in the human capital value of young people. This finding suggests that interventions for younger women can be an important political challenge. Several studies on the economic burden of cervical cancer have been conducted in other countries. For example, Ricciardi et al. [24] estimated the annual DC of managing invasive cancer in Italy as 28.3 million Euro per year. In 2003, Olivia et al. [25] estimated the IC of cervical cancer in Spain using two alternative approaches (human capital method and friction cost method). The annual IC was estimated as 43.4 million Euro by the human capital method and 1.1 million Euro by the friction cost method. However, no such long-term studies have been conducted, and none have made future predictions. Therefore, this is one of the advantages of our study.

Routine prophylactic vaccination against cervical cancer is currently being introduced in Japan. Prophylactic vaccination against cervical cancer is expected to further reduce the number of patients with cervical cancer and related deaths, which should contribute to reducing the COI.

A limitation of the present study is the fact that the COI method only analyzes macroestimates of costs without taking into account the quality of medical care or quality of life, and it does not involve verification of the cost effectiveness of microlevel therapeutic techniques. Moreover, to who and what these costs apply is unclear. For example, several studies have reported the economic effects of prophylactic vaccination against human papillomavirus [3,26-28], and prophylactic vaccination may help to reduce the healthcare costs associated with cervical cancer. Our study does not take the effect of a specific treatment technology into consideration; a further study of COI that includes the possible effect of prophylactic vaccination against cervical cancer treatment would be needed. In addition, because the annual labor value is fixed at the 2011 value, as women’s participation in society continues to accelerate and their incomes increase, MbC and MtC are likely to be underestimated using this method. However, the COI method enables the support of rational decision-making through the efficient use of limited medical resources by measuring the monetary value of disease burden [6,7]. Furthermore, this method provides basic information on policy-making related to prevention and management activities for injuries and diseases that justify intervention plans [10]. Therefore, the knowledge obtained from the present study is valid and can be applied to future policy-making.

## Conclusions

We made future predictions of the COI of cervical cancer using government statistics. According to our mixed estimates, which are considered highly valid, if the current trends in health-related indicators continue, the COI will temporarily decrease in 2014, followed by almost no change until 2020. The lack of a prolonged life expectancy in individuals with cervical cancer and the lack of a decrease in the number of deaths among young women with a high human capital value are likely factors preventing a future reduction in the COI. Interventions involving young women have high political priority because the COI has high possibility to increase in the future secondary to the promotion of women’s participation in society.

## Additional file

Additional file 1:
**Time trend of total person-days of outpatient visits (TOVy), total person-days of hospitalization (THD), and lifetime labor value per person (LVl).**

## Abbreviations

COI: Cost of illness
DC: Direct cost
IC: Indirect cost
MbC: Morbidity cost
MtC: Mortality cost
ENP: Estimated number of patients
LVd: 1-day labor value per person
LVl: Lifetime labor value per person
TOVy: Total person-days of outpatient visits
THD: Total person-days of hospitalization
NDy: Number of deaths
Iy: Annual income per person
ULVy: Annual monetary valuation of unpaid work per person
HPy: Annual number of hospitalized patients
ALOS: Average length of stay
